# Soil Microbial Communities Affect the Growth and Secondary Metabolite Accumulation in *Bletilla striata* (Thunb.) Rchb. f.

**DOI:** 10.3389/fmicb.2022.916418

**Published:** 2022-06-06

**Authors:** Chenghong Xiao, Chunyun Xu, Jinqiang Zhang, Weike Jiang, Xinqing Zhang, Changgui Yang, Jiao Xu, Yongping Zhang, Tao Zhou

**Affiliations:** ^1^Resource Institute for Chinese and Ethnic Materia Medica, Guizhou University of Traditional Chinese Medicine, Guiyang, China; ^2^College of Pharmacy, Guizhou University of Traditional Chinese Medicine, Guiyang, China

**Keywords:** *Bletilla striata*, soil microbiome, secondary metabolites, sandy clay soil, sandy loam soil

## Abstract

*Bletilla striata* (Thunb.) Rchb.f. is a perennial herb belonging to the Orchidaceae family. Its tubers are used in traditional Chinese medicine to treat gastric ulcers, inflammation, silicosis tuberculosis, and pneumogastric hemorrhage. It has been reported that different soil types can affect the growth of *B. striata* and the accumulation of secondary metabolites in its tubers, but the biological mechanisms underlying these effects remain unclear. In this study, we compared agronomic traits and the accumulation of secondary metabolites (extractum, polysaccharide, total phenol, militarine) in *B. striata* grown in sandy loam or sandy clay soil. In addition, we compared physicochemical properties and microbial communities between the two soil types. In pot experiments, we tested how irradiating soil or transplanting microbiota from clay or loam into soil affected *B. striata* growth and accumulation of secondary metabolites. The results showed that sandy loam and sandy clay soils differed significantly in their physicochemical properties as well as in the structure and composition of their microbial communities. Sandy loam soil had higher pH, SOM, SOC, T-Ca, T-N, T-Mg, T-Mn, T-Zn, A-Ca, A-Mn, and A-Cu than sandy clay soil, but significantly lower T-P, T-K, T-Fe, and A-P content. Sandy loam soil showed 7.32% less bacterial diversity based on the Shannon index, 19.59% less based on the Ace index, and 24.55% less based on the Chao index. The first two components of the PCoA explained 74.43% of the variation in the bacterial community (PC1 = 64.92%, PC2 = 9.51%). Similarly, the first two components of the PCoA explained 58.48% of the variation in the fungal community (PC1 = 43.67%, PC2 = 14.81%). The microbiome associated with sandy clay soil can promote the accumulation of militarine in *B. striata* tubers, but it inhibits the growth of *B. striata.* The accumulation of secondary metabolites such as militarine in *B. striata* was significantly higher in sandy clay than in sandy loam soil. Conversely, *B. striata* grew better in sandy loam soil. The microbiome associated with sandy loam soil can promote the growth of *B. striata*, but it reduces the accumulation of militarine in *B. striata* tubers. Pot experiment results further confirmed that the accumulation of secondary metabolites such as militarine was higher in soil transplanted with loam microbiota than in soil transplanted with clay microbiota. These results may help guide efforts to improve *B. striata* yield and its accumulation of specific secondary metabolites.

## Introduction

Plants (including orchids) can produce a variety of secondary metabolites, which not only play an important role in plant defense against pathogenic attack and environmental stress (Yang et al., 2018), but also have important pharmacological and physiological activities (Wink, 2015). *Bletilla striata* (Thunb.) Rchb.f. is a perennial herb belonging to the Orchidaceae family. It has high ornamental value (Xi et al., 2020), and its tubers are used in traditional Chinese medicine to treat gastric ulcers, inflammation, silicosis tuberculosis and pneumogastric hemorrhage (Zhang et al., 2021; Zhou et al., 2021). Several studies have confirmed that the anti-inflammatory, antioxidant, antibacterial, and anti-tumor effects exhibited by *B. striata* (Li et al., 2018; Xu et al., 2019; Liang et al., 2021) occur as a result of its secondary metabolites, especially extractum, polysaccharides phenolic compound, and militarine present in the tubers (Jiang et al., 2013). Therefore, it is of great significance to improve the accumulation of secondary metabolites in plants.

Orchids are typically unable to obtain nutrients directly from the soil. Therefore, microbiome associations play an important role in their life cycle, especially among orchids grown under natural conditions (Zhao X. et al., 2014). Root bacteria that promote plant growth are beneficial bacteria for root colonization. They can facilitate plant growth directly by either assisting in resource acquisition (nitrogen, phosphorus, and essential minerals) or modulating plant hormone levels, or indirectly by inhibiting various pathogens as biocontrol agents (Haque et al., 2020). As well as, they can mitigate abiotic stresses, including water-deficit stress and salt stress (Haque et al., 2022). Studies have found that application of *Bacillus amyloliquefaciens* and *Paraburkholderia fungorum* not only can significantly increase growth and fruit yield but also enhance functional properties of strawberry by inducing enhanced production of total antioxidants, carotenoids, favonoids, phenolics, and anthocyanins (Rahman et al., 2018). In addition, mycorrhizal fungi are typically regulated by characteristics of the soil, including their physical and chemical properties as well as microbial composition and by characteristics of the plant community (Mujica et al., 2016; Ma et al., 2018).

Soil health refers to the ability to support and sustain crop growth and productivity, and a key component of it is the abundance of plant growth-promoting organisms, such as root bacteria (Khatoon et al., 2020). It has been reported that different soil types can have different effects on plant growth based on their water, nutrient availability and soil microbiology (Ozaslan et al., 2016). Studies have found that soil type and cultivar influenced the available nitrogen, phosphorus, potassium, and organic carbon content of rhizosphere soil, and rhizosphere microbial communities (Sneha et al., 2021). And soil microbial community plays an important role in the accumulation of plant secondary metabolites (Salla et al., 2016). Whether and how these factors affect the growth and accumulation of secondary metabolites in *B. striata* tubers remains unclear. In this study, we examined the effects of soil type and soil microbial communities on the growth and accumulation of secondary metabolites in *B. striata* tubers.

## Materials and Methods

### Experiments

Field and pot experiments were conducted using 1-year-old *B. striata* tissue culture seedlings (Guizhou Fusheng Tianhong Ecological Industry Development, Guiyang, China). The soil samples of *B. striata* in Guizhou province were classified based on the US Department of Agriculture Soil Taxonomy guidelines (Bame et al., 2013). In Guizhou, China, *B. striata* resources mostly grow in sandy loam and sandy clay soil. Therefore, two different types of soil were identified and collected in Guizhou, China (26°57′52″N, 108°1′33″E).

#### Field Experiments

In March 2017, field experiments were performed in Shibing in Guizhou, China. A total of thirty *B. striata* tissue culture seedlings according to the spacing of 20 cm were planted in sandy loam or sandy clay soil, during the growing period, soil moisture should be kept at 60 ∼ 80%, timely watering and drainage, apply fertilizer once in April and October every year, 12 g compound fertilizer was applied to each plant. The climate in this region is mild and humid, with weak solar radiation, annual sunshine of about 1,200 h, average altitude of about 800 m, average annual temperature of 14 ∼ 16°C, frost-free period of 255 ∼ 294 days, and annual precipitation of 1,060 ∼ 1,200 mm.

#### Pot Experiments

In April 2019, sandy loam and sandy clay soil samples were collected from the same location where the field experiments were performed. Each soil sample was then divided into two parts, one of which was subjected to 25 kGy cobalt-60 γ -ray irradiation sterilization for 3 days, the other is not irradiation. The mother soil which consisted of nutrient soil, loam, coconut brick, and vermiculite in a volume ratio of 3:1:1:1. The nutrient soil organic matter content was 45%, and the nitrogen, phosphorus, and potassium content were 6% (Dewoduo Fertilizer Co., LTD., Hengshui, China). The mother soil organic matter content was 22.5%, and the nitrogen, phosphorus, and potassium content were 3.02, 3.01, and 3.09%, respectively. The mother soil was also subjected to irradiation.

The experimental procedure involved the following three treatments: (1) clay-microbiota transplanted soil (CMTS), each pot consisting of irradiated mother soil (1,000 mL), irradiated sandy loam soil (50 mL), and non-irradiated sandy clay soil (50 mL); (2) loam-microbiota transplanted soil (LMTS), each pot consisting of irradiated mother soil (1,000 mL), non-irradiated sandy loam soil (50 mL), and irradiated sandy clay soil (50 mL); and a control group (CK), each pot consisting of irradiated mother soil (1,000 mL), irradiated sandy loam soil (50 mL), and irradiated sandy clay soil (50 mL). Thirty pot replicates were performed for each treatment. All pots are sized at 18 cm in diameter, 11 cm in height, and 10 cm in bottom diameter.

A total of ninety *B. striata* tissue culture seedlings with similar plant height were selected, tubers and roots were soaked in 0.1% mercury disinfectant for 10 min, then rinse with sterilized water and planted in the center of flower pots at the same depth, with about 2 mm of soil covering the tuber. We planted one seedling in each pot, and the pots were placed in a greenhouse in Guizhou, China. Each treatment was administered to a set of thirty pots that were placed in random locations and watered each week. The plants were covered with plastic film to prevent bacterial contamination and on their requirements.

### Analyses of Plants

#### Samples

In the field experiment, *B. striata* samples were harvested 2 years after planting (in March 2019), and five samples were randomly selected for analysis. The preliminary experiment found that there were differences in the growth and accumulation of secondary metabolites of *B. striata* after 6 months treatment. Therefore, in the pot experiment, *B. striata* samples were collected 6 months after planting (in October 2019), and five samples corresponding to each treatment were randomly selected for further analysis.

#### Agronomic Traits

Before collecting the *B. striata* samples, we measured plant height, leaf width, and leaf length, and we recorded the numbers of stems, leaves, and flowers. Plants collected from the field and the pots were dried in an oven at 50°C to a constant weight (Zhang et al., 2022). We measured dry weight aboveground and underground (tuber and fibrous roots) using an electronic balance (Mettler Toledo, Shanghai, China). These parameters were used to evaluate the effects of two soil microbial communities on *B. striata* growth.

#### Extractum Content

Plant extractum content was determined using the cold immersion method. Each accurately weighed sample (2 g) was placed in a 150 mL conical flask containing 50 mL of 50% (v/v) ethanol. The flask was then soaked in cold water and shaken frequently for the first 6 h, after which it was left undisturbed for 18 h before straining. Next, 20 mL of the filtrate was collected and dried in an evaporation dish until a constant weight was achieved. After being steamed in a water bath for 1 h and dried at 105°C for 3 h, the sample was cooled in a dryer for 30 min and weighed immediately.

#### Militarine Content

Militarine content in the samples was determined using high-performance liquid chromatography (Waters, Milford, Massachusetts, United States) (Liu et al., 2019). After accurately weighing the reference substance (militarine), methanol was added to prepare a 0.599 mg/mL solution of the reference substance. *B. striata* samples were ground into a powder and accurately weighed (0.05 g), then placed in a 10 mL centrifuge tube containing methanol (3 mL). The samples were subjected to ultrasonic treatment for 1 h (100 W, 40 Hz), after which the cooled solution was filtered through a 0.45-μm filter. The following chromatographic conditions were used: column, thermore C_18_ (250 mm × 4.6 mm, 5 μm); detection wavelength, 223 nm; column temperature, 40°C; flow rate, 1 mL/min; injection volume, 5 μL. The mobile phase consisted of water and methanol, and the methanol gradient was as follows: from 0 to 2 min, the methanol concentration increased from 37 to 40%; 2–5 min, from 40 to 45%; and 5–15 min, from 45 to 50%. The methanol concentration remained at 50% from 15 to 20 min.

#### Total Phenol Content

Plant total phenol Tp ELISA Kit was performed according to the manufacturer’s instructions (Fankel, Shanghai, China) to determine the total phenol content in *B. striata* samples. Samples were analyzed at 450 nm using a microplate reader (Thermo Fisher Scientific, Wilmington, DE, United States).

#### Polysaccharide Content

Water extraction and alcohol precipitation (Zheng et al., 2014) were used to prepare *B. striata* polysaccharide solutions for analysis. Briefly, dried *B. striata* samples (0.05 g) were extracted once with water (3 mL) for 2 h to obtain a water extractum. Ethanol was used to precipitate the polysaccharides from the aqueous solution, which were redissolved in water. A standard reference solution was prepared by accurately weighing 0.02 g of anhydrous D-glucose (National Institutes for Food and Drug Control, Beijing, China) and dissolving it in 25 mL of water in a 100 mL volumetric flasks. This reference stock solution was then diluted to various concentrations (10.0, 50.0, 100.0, 150.0, and 200.0 μg/mL). Polysaccharide content was determined by using anthrone-sulfuric acid as the chromogenic agent and measuring absorbance at 625 nm using a UV-2700 ultraviolet spectrophotometer (Shimadzu, Kyoto, Japan).

### Analyses of Soil

#### Soil Samples

Five samples of sandy loam and five samples of sandy clay corresponding to the soil in the field samples were collected at a depth of 10–15 cm, placed into re-sealable plastic bags and stored at 4°C until analysis of physicochemical properties. Microbial analysis was performed on rhizosphere soil, defined as the soil tightly attached to the root surface: samples were placed in sterile plastic bags and stored at −80°C in the laboratory. Both loose and rhizosphere soil samples were collected using the double diagonal five-point sampling method. In briefly, the midpoint of the diagonal is first determined as the central sampling point, and then four points on the diagonal with equal distance from the central sample point are selected as sample points. After removing topsoil plant residue, stones and other debris, the samples were air-dried and sieved to obtain a fraction that was ≤ 2 mm.

#### Analysis of Soil Physicochemical Properties

Soil organic carbon (SOC) content was determined using the Kaushal Method (Kaushal et al., 2020), while soil organic matter (SOM) content was determined using the H_2_SO_4_-K_2_Cr_2_O_7_ oxidation method (Wenjun et al., 2014). Soil pH was measured in soil suspensions (1:2.5) using a pH electrode (Bi et al., 2020). Total nitrogen (T-N) content was determined using the Kjeldahl Method (Roth et al., 2014), while ammonium nitrogen content in a KCl (2 mol/L) extract was measured using the indophenol blue colorimetric method (Ye et al., 2017). Total phosphorus (T-P) and available phosphorus (A-P) concentrations were measured using an UV-2700 ultraviolet spectrophotometer (Hou et al., 2020), while the concentration of total potassium (T-K), total calcium (T-Ca), and total magnesium (T-Mg) were measured using flame photometry (Mishra et al., 2020). Available iron (A-Fe), available manganese (A-Mn), and available zinc (A-Zn) were extracted with a solution containing nitric acid, hydrogen peroxide, and hydrofluoric acid in a 6:2:2 ratio. Available copper (A-Cu) was extracted using diethylene triamine penta-acetic acid (Manzeke et al., 2019). After these extractions, flame photometry was used to measure total iron (T-Fe), total manganese (T-Mn), total zinc (T-Zn), and total copper (T-Cu) concentrations. The same method was applied again to measure available calcium (A-Ca), available potassium (A-K), and available magnesium (A-Mg) concentrations after extraction with ammonium acetate (NH_4_OAc, 1 mol/L, pH = 7) (Bi et al., 2020).

#### Soil Microbial Community Composition

High-throughput MiSeq sequencing was performed (Majorbio, Shanghai, China) in order to identify the microbial species composition of the sandy loam and sandy clay soil samples. The original data can be downloaded from “https://submit.ncbi.nlm.nih.gov/subs/bioproject/SUB11365206/overview” website. Sequencing experiments involved the following steps: DNA extraction, PCR amplification and product purification, real-time quantitative PCR, MiSeq library construction, and MiSeq sequencing. TransStart FastPfu DNA Polymerase (TransGen Biotech, Beijing, China) was used for PCR amplification, which was carried out using the ABI GeneAmp^®^ 9700 (Thermo Fisher Scientific, Wilmington, DE, United States). Fungal DNA fragments were amplified using the ITS1-forward primer (5′-GGAAGTAAAAGTCGTAACAAGG-3′) and the ITS2-reverse primer (5′-ATCCTCCGCTTATTGATATGC-3′), while bacterial DNA fragments were amplified using the 338-forward primer (5′-GTACTCCTACGGGAGGCAGCA-3′) and the 806-reverse primer (5′-GTGGACTACHVGGGTWTCTAAT-3′). DNA concentration and purity were measured in 1% agarose gels using a NanoDrop 2000 UV-vis spectrophotometer (Thermo Fisher Scientific, Wilmington, DE, United States).

### Statistical Analyses

All statistical analyses were performed using GraphPad Prism 8.0 (Graphpad, Chicago, United States). Data were presented as mean ± standard error of the mean (SEM) (*n* = 5). Pairwise comparisons were assessed for significance using Student’s two-tailed *t*-tests, and comparisons among three or more values were assessed using one-way ANOVA and Tukey’s *post hoc* test. The following *p*-values were considered to indicate significance: *p* < 0.05, significant; *p* < 0.01, very significant; and *p* < 0.001, highly significant.

## Results

### Effects of Soil Type on Growth and Secondary Metabolite Content in *Bletilla striata*

**FIGURE 1:**
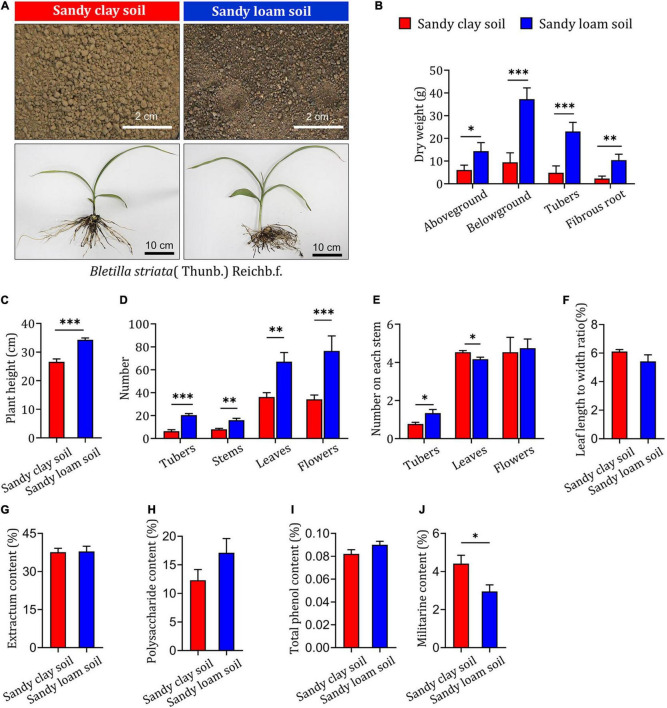
Impact of soil type on growth and secondary metabolite content in *B. striata*. **(A)** Representative image of sandy clay soil (top left, red bars) and sandy loam soil (top right, blue bars) and *Bletilla striata* (Thunb.) Rchb.f. plants grown in these soils. Scale bars: 2 or 10 cm. **(B)** Dry weight of aboveground biomass, belowground biomass, tubers, and fibrous roots of *B. striata* planted in sandy clay or sandy loam soils. **(C)** Height of *B. striata* planted in sandy clay or sandy loam soils. **(D)** Numbers of tubers, stems, leaves, and flowers of *B. striata* planted in sandy clay or sandy loam soils. **(E)** Numbers of tubers, stems, leaves, and flowers on each stem of *B. striata* planted in sandy clay or sandy loam soils. **(F)** Ratio of leaf length to width in *B. striata* planted in sandy clay or sandy loam soils. Content of **(G)** extractum, **(H)** polysaccharides, **(I)** total phenols, and **(J)** militarine in *B. striata* planted in sandy clay and sandy loam soils. Data are mean ± SEM (*n* = 5). *p < 0.05, ^**^p < 0.01, and ^***^p < 0.005 based on the independent-sample *t*-test.

To assess the impact of soil type on the growth and development of *B. striata*, field experiments were carried out in sandy loam and sandy clay soils. Samples were collected and tested 2 years after planting (Figure 1A). *B. striata* seedlings planted in sandy loam soil were taller and had higher aboveground, belowground, tuber, and fibrous root dry weights than those grown in sandy clay soil (Figures 1B,C). They also had significantly higher numbers of tubers, stems, leaves, flowers, and tubers on each stem than seedlings grown in sandy clay soil. The opposite trend was observed with respect to the number of leaves on each stem. There was no significant difference between seedlings planted in sandy clay and sandy loam soil in terms of the number of flowers on each stem or the ratio of leaf length to width (Figures 1D–F).

Our analysis of extractum, polysaccharide, and total phenol content showed no significant differences between *B. striata* seedlings planted in sandy loam or sandy clay soils (Figures 1G–I). In contrast, militarine content was significantly higher in seedlings planted in sandy clay soil (Figure 1J). These results indicate that sandy loam soil is associated with reduced synthesis of secondary metabolites and increased growth in *B. striata*. Sandy clay soil, in contrast, is more conducive to the synthesis of secondary metabolites and can inhibit the growth of *B. striata.*

### Differences in Physicochemical Properties of Sandy Clay and Sandy Loam Soils

**FIGURE 2:**
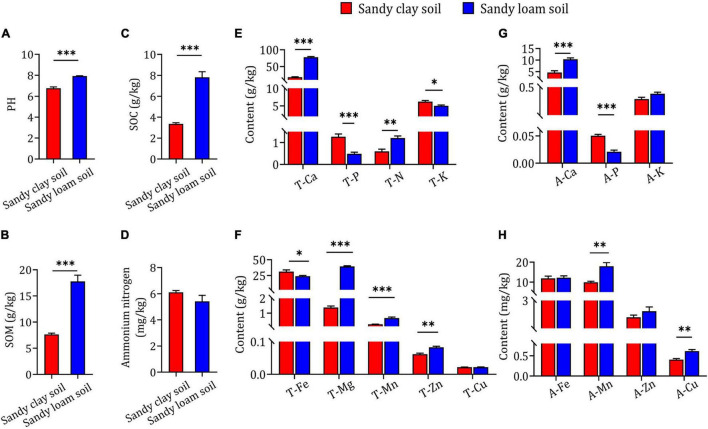
Physicochemical properties and elemental concentrations in sandy clay and sandy loam soils. **(A)** Soil pH, **(B)** soil organic matter (SOM), **(C)** soil organic carbon (SOC), and **(D)** ammonium nitrogen concentration in sandy clay soil (red bars) and sandy loam soil (blue bars). **(E,F)** Total nutrient element content in sandy clay and sandy loam soils: total calcium (T-Ca), total phosphorus (T-P), total nitrogen (T-N), total potassium (T-K), total iron (T-Fe), total magnesium (T-Mg), total manganese (T-Mn), total zinc (T-Zn), and total copper (T-Cu). **(G,H)** Available element content in sandy clay and sandy loam soils: available calcium (A-Ca), available phosphorus (A-P), available potassium (A-K), available iron (A-Fe), available manganese (A-Mn), available zinc (A-Zn), and available copper (A-Cu). Data are mean ± SEM (*n* = 5). *p < 0.05, ^**^p < 0.01, and ^***^p < 0.005 based on the independent-sample *t*-test.

In order to confirm whether the differences in *B. striata* growth and secondary metabolite content occurred as a result of differences in soil type, we evaluated the physicochemical properties of the soils. We found that sandy loam soil had higher pH, SOM and SOC than sandy clay soil (Figures 2A–C). There was no significant difference between the two soil types in ammonium nitrogen content (Figure 2D). Additionally, sandy loam soil had significantly higher T-Ca, T-N, T-Mg, T-Mn, T-Zn, A-Ca, A-Mn, and A-Cu content than sandy clay soil, but significantly lower T-P, T-K, T-Fe, and A-P content. There were no significant differences between the two soil types in T-Cu, A-K, A-Fe, or A-Zn content (Figures 2E–H). These results indicate that sandy loam and sandy clay soils differ significantly in physicochemical properties.

### Effects of Soil Type on Microbial Communities

**FIGURE 3:**
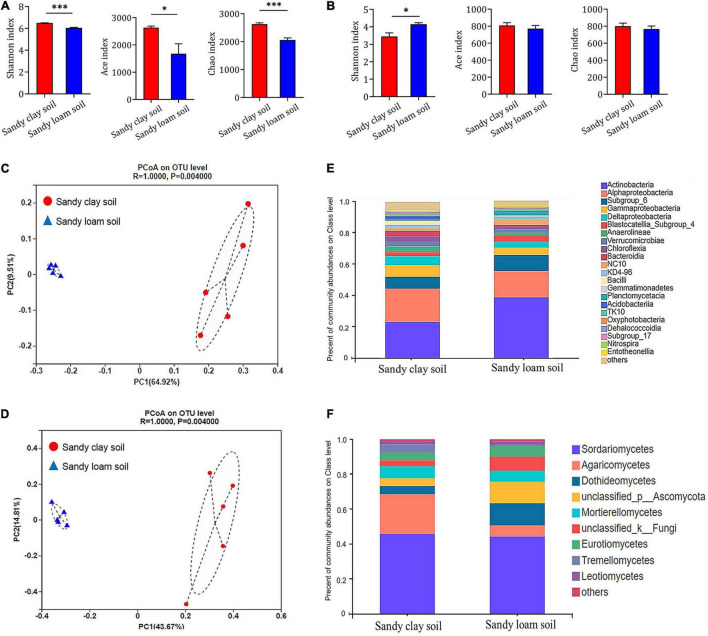
Composition and structure of microbial communities in sandy clay and sandy loam soils. **(A,B)** Alpha diversity at the level of OTUs in **(A)** bacterial and **(B)** fungal communities in sandy clay soil (red) and sandy loam soil (blue). Microbial diversity was quantified using Chao’s, Ace’s, and Shannon’s diversity indices (*n* = 5). **(C,D)** Principal component analysis (PCoA) of beta diversity in **(C)** bacterial and **(D)** fungal communities in sandy clay and sandy loam soils, based on Bray-Curtis distances. **(E,F)** Relative abundances of **(E)** bacteria and **(F)** fungi in sandy clay and sandy loam soils. Data are mean ± SEM (*n* = 5). *p < 0.05 and ^***^p < 0.005 based on the independent-sample *t*-test.

We used various indices to assess microbial species diversity in the two soil types. Based on Shannon’s, Ace’s, and Chao’s diversity indices, we found that the bacterial community in sandy clay soil was significantly more diverse than that in sandy loam soil at the level of operational taxonomic units (OTUs). Sandy loam soil showed 7.32% less bacterial diversity based on the Shannon index, 19.59% less based on the Ace index, and 24.55% less based on the Chao index (Figure 3A).

Similarly, the fungal community in sandy clay soil showed higher species diversity at the level of OTUs than that in sandy loam soil based on Ace’s and Chao’s diversity indices, but these differences were not significant. Conversely, the fungal community in sandy clay soil showed lower Shannon diversity than that in sandy loam soil (Figure 3B).

Using principal coordinate analyses (PCoA) based on Bray-Curtis distance, we detected differences in bacterial and fungal community structure between the two soil types. The first two components of the PCoA explained 74.43% of the variation in the bacterial community (PC1 = 64.92%, PC2 = 9.51%; Figure 3C). Similarly, the first two components of the PCoA explained 58.48% of the variation in the fungal community (PC1 = 43.67%, PC2 = 14.81%; Figure 3D). When we analyzed the relative abundance of the bacterial and fungal communities at the class level, we found that soil microorganisms such as *Alphaproteobacteria*, *Gammaproteobacteria*, *Deltaproteobacteria*, and *Agaricomycetes* had higher relative abundance in sandy clay soil than in sandy loam soil. However, the opposite trend was observed in the case of *Actinobacteria* and *Dothideomycetes* (Figures 3E,F).

**FIGURE 4:**
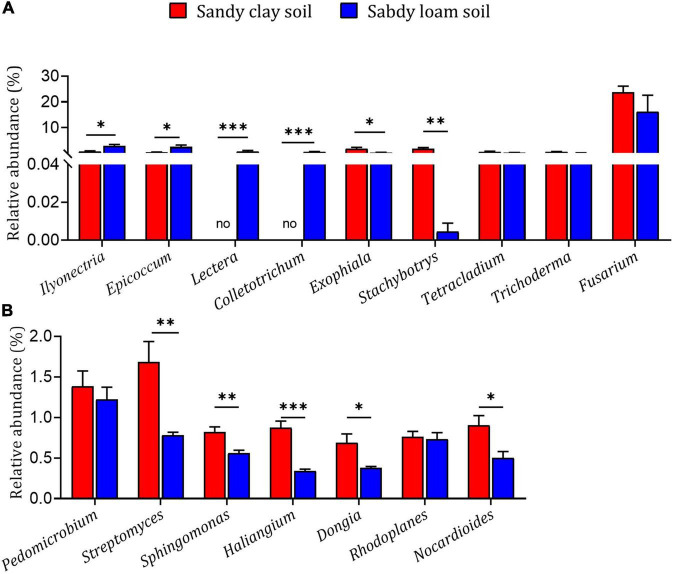
Relative abundances of microbial genera in sandy loam and sandy clay soils. Relative abundances of **(A)** fungal genera and **(B)** bacterial genera in sandy loam and sandy clay soils. Data are mean ± SEM (*n* = 5). *p < 0.05, ^**^p < 0.01, and ^***^p < 0.005 based on the independent-sample *t*-test.

The fungal genera *Ilyonectria*, *Epicoccum*, *Lectera*, and *Colletotrichum* had higher relative abundance in sandy loam soil than in sandy clay soil, while the genera *Exophiala* and *Stachybotrys* had lower relative abundance in sandy loam soil (Figure 4A). The bacterial genera *Streptomyces*, *Sphingomonas*, *Haliangium*, *Dongia*, and *Nocardioides* had higher relative abundance in sandy clay soil than in sandy loam soil (Figure 4B). These results indicate that microbial diversity and relative abundance can differ significantly between the two soil types.

### Impact of Microbial Community on Growth and Secondary Metabolite Content in *Bletilla striata*

**FIGURE 5:**
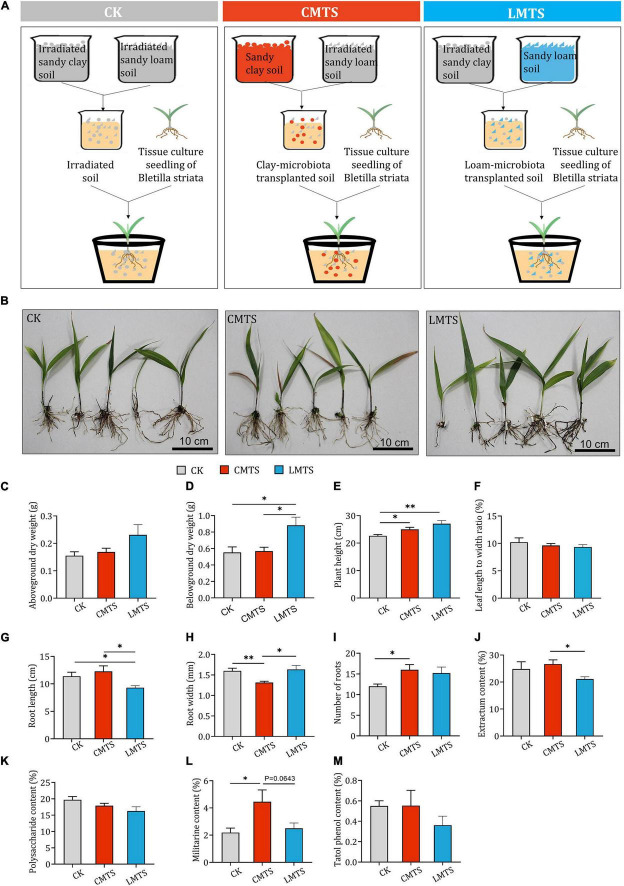
Effects of microbial community on growth and secondary metabolite content in *B. striata*. **(A)** Schematic depicting planting of *B. striata* seedlings in experimental pots containing irradiated soil (CK), clay-microbiota transplanted soil (CMTS), or loam-microbiota transplanted soil (LMTS). **(B)** Representative photographs of *B. striata* plants grown under the three conditions. **(C–I)** Post-treatment quantification of the following *B. striata* parameters: **(C)** Aboveground dry weight, **(D)** belowground dry weight, **(E)** height, **(F)** ratio of leaf length to width, **(G)** number of roots, **(H)** root length, and **(I)** root width. **(J–M)** Post-treatment quantification of the following *B. striata* parameters: **(J)** Extractum content, **(K)** polysaccharide content, **(L)** militarine content, and **(M)** total phenol content in *B. striata*. Data are mean ± SEM (*n* = 5). *p < 0.05 and **p < 0.01 based on the independent-sample *t*-test.

To assess the impact of different microbial communities on the growth and development of *B. striata*, pot experiments were carried out using irradiated soil (CK group), loam-microbiota transplanted soil (LMTS group), and clay-microbiota transplanted soil (CMTS group; Figure 5A). Samples of *B. striata* were collected from each group at 6 months after planting (Figure 5B) and processed for further analyses. We found that the aboveground dry weight of *B. striata* was higher in the LMTS and CMTS groups than in the CK group, but these differences were not significant (Figure 5C). The belowground dry weight of *B. striata* was significantly higher in the LMTS group than in the CK and CMTS groups (Figure 5D). *B. striata* plants in the LMTS and CMTS groups were significantly taller than those in the CK group (Figure 5E). There was no significant difference among the three groups in the ratio of leaf length to width (Figure 5F). Root length was significantly higher in the CK and CMTS groups than in the LMTS group (Figure 5G), while root width was significantly lower in the CMTS group than in the CK and LMTS groups (Figure 5H). In addition, the number of roots was higher in the CMTS and LMTS groups than in the CK group (Figure 5I).

Plant extract content was significantly higher in *B. striata* in the CMTS group than those in the LMTS group. Although there was no significant differences in militarine content between CMTS group and LMTS group (p=0.0643), militarine content was higher in *B. striata* in the CMTS group than those in the LMTS group. There were no significant differences in polysaccharide or total phenol content among the three treatments (Figures 5J–M).

These results indicate that the microbial community in sandy loam soil can be beneficial for *B. striata* growth and lead to a reduction in the synthesis of secondary metabolites. On the other hand, the microbial community in sandy clay soil favors the accumulation of secondary metabolites and can inhibit the growth of *B. striata*.

## Discussion

*B. striata* plays an important role in traditional Chinese medicine (Xi et al., 2020), and its chemical composition and medicinal properties have been studied extensively (Xu et al., 2019). The growth of *B. striata* is affected by many factors, among which soil type is one of the most important factors. However, very little is known about the effects of soil type on *B. striata* growth and the accumulation of secondary metabolites in their tubers. In the present study, we tested the effects of sandy loam and sandy clay soils, and the microbial communities within those soils, on growth and secondary metabolite content in *B. striata*. We observed significant differences between the two soil types in physicochemical properties, as well as in the soil microbial communities that they supported. These differences can have a significant impact on the growth of *B. striata.*

Our results indicate that microorganisms such as *Streptomyces* that are present in sandy clay soil may promote the accumulation of militarine and other secondary metabolites in *B. striata*. Sandy loam soil, in contrast, may not be conducive to the accumulation of secondary metabolites, yet it contains microorganisms such as *Colletotrichum* that may promote *B. striata* growth. These findings may help guide efforts to promote *B. striata* growth and accumulation of specific secondary metabolites.

### Sandy Loam and Sandy Clay Soils Have a Significant Impact on the Growth and Development of *Bletilla striata*

Different soil types can have different effects on plant growth based on their water and nutrient availability (Ozaslan et al., 2016). For example, *Jatropha curcas* L. grows better in sandy and loamy soils than in cohesive soils (Valdés-Rodríguez et al., 2013). Similarly, soil texture can significantly affect crop yield. For example, rice grain yields are 46% higher in clay soil than in sandy soil (Dou et al., 2016). Grape plants show faster root growth and higher total anthocyanin content in sandy soil than in clay soil (Leibar et al., 2017).

Militarine is one of the major secondary metabolites and medicinally active components of *B. striata*, and can exhibit anti-inflammatory, anti-oxidant, and anti-apoptotic effects (Tian et al., 2021). Our results suggest that although sandy loam soils promote the growth of *B. striata*, they also reduce the accumulation of militarine in *B. striata* tubers. In contrast, sandy clay soils can inhibit the growth of *B. striata*, as well as increase the accumulation of militarine in the tubers.

### Soil Elemental Composition Can Affect *Bletilla striata* Growth and the Accumulation of Secondary Metabolites

Organic carbon content, elemental nutrient concentrations, and base status differ significantly across soil types (Bame et al., 2013), and these differences can influence plant growth and development. In a study conducted in Hamedan, western Iran, the organic matter content in sandy loam and mixed loam soil was 2.3%, while sandy soil contained only 0.4% organic matter (Jalali and Jalali, 2016). Previous studies have reported that clay soil is more likely to bind phosphorus than sandy soil (Andersson et al., 2013). Since calcium, magnesium, nitrogen, potassium, and phosphorus are essential macronutrients for plant growth and development, their availability directly affects crop yield (Wang et al., 2021). For example, increased availability of calcium and magnesium can promote growth in rice crops (Weijie et al., 2021). In the present study, we found significantly higher calcium and magnesium content in sandy loam soil than in sandy clay soil, which may help explain the better growth of *B. striata* in sandy loam soil.

Levels of phosphorus, manganese, zinc, and other nutrients are closely related to the synthesis of secondary metabolites (Jia et al., 2016). Excessive or limiting levels of phosphorus can reduce the accumulation of flavonoids in tea leaves, thus weakening the anti-oxidant activity of tea, but the rhizosphere usually cannot provide sufficient available phosphorus to satisfy plant demands (Ding et al., 2017). In the present study, we found that sandy loam soils contained significantly higher concentrations of phosphorus than sandy clay soils, which may help explain the greater accumulation of militarine in *B. striata*. Further research must be conducted to gain a better understanding of this relationship.

### Variation in Soil Microbial Communities Can Affect *Bletilla striata* Growth and the Accumulation of Secondary Metabolites in Tubers

Soil type can affect plant growth by determining nutrient availability (Ozaslan et al., 2016) and microbial community structure (Sun et al., 2015). There is a complex community relationship between soil microorganisms and plants. Soil microorganisms are essential for plant growth and survival, and plants can also influence soil microbial communities through secondary metabolites (Siebers et al., 2018). Based on the Chao’s, Ace’s, and Shannon’s diversity indices, sandy clay soils had significantly higher bacterial diversity than sandy loam soils. These results suggest that *B. striata* growing in sandy clay may produce more secondary metabolites benficial to soil microorganisms but further verification is needed.

Soil microbial communities are affected by soil physicochemical properties, especially total soil carbon and SOC (Qu et al., 2018). Mulberry/alfalfa intercropping can change soil bacterial communities by altering SOC, A-P, and A-K (Zhang et al., 2018). *Colletotrichum tofieldiae* has been shown to colonize *Arabidopsis* roots and transfer phosphorus to it, promoting plant growth and increasing fertility under phosphate-deficient conditions (Hiruma et al., 2016). *Trichoderma asperellum* strains can mobilize insoluble iron to provide mineral nutrients for plant growth (Zhao L. et al., 2014), while *Streptomyces* sp. can substantially increase the uptake and translocation of phosphorus (Battini et al., 2017). The endophytic fungus *Fusarium oxysporum*, which colonizes the roots of *B. striata*, can promote seed germination (Jiang et al., 2019). In the present study, *Trichoderma*, *Streptomyces*, *Fusarium*, and *Epicoccum* were observed in sandy loam and sandy clay soil, while *Colletotrichum* was detected only in sandy loam soil. This suggests that improved growth of *B. striata* in sandy loam soil may be because *Colletotrichum* promotes the absorption of phosphorus, thus promoting the growth of *B. striata*. Further research must be conducted to validate and extend our results.

Secondary metabolites promote healthy growth of plants and are useful as active ingredients in traditional Chinese medicine (Battini et al., 2016). *Streptomyces* can promote the accumulation of phenolic compounds and flavonoids in *Eucalyptus grandis* shoots, as well as alter enzymatic activities and phenolic content in *E. globulus* (Salla et al., 2014). In fact, *Streptomyces* sp. PM9 can increase basal levels of two enzymes and synthesis of phenolics that contribute to disease responses in *E. grandis* (Salla et al., 2016).

In the present study, we found that the relative abundance of *Streptomyces* in sandy clay soil was significantly higher than that in sandy loam soil. Therefore, we speculate that *Streptomyces* promotes the synthesis of specific secondary metabolites in *B. striata.* In support of this, we found that *B. striata* planted in CMTS plots had significantly higher militarine levels than plants in LMTS pots. Conversely, LMTS plants showed greater belowground biomass and thicker roots. Our findings indicate that the presence of microorganisms such as *Colletotrichum* in sandy loam soil are more beneficial to *B. striata* growth, while microorganisms such as *Streptomyces* in sandy clay soil are beneficial for the accumulation of secondary metabolites in *B. striata.*

## Conclusion

Our results suggest that variation in soil microbial communities as a result of differences in soil physicochemical properties and elemental concentrations can affect *B. striata* growth and the accumulation of secondary metabolites in tubers. Although the microbiome in sandy loam soils can promote the growth of *B. striata*, it can reduce the accumulation of militarine in tubers. Conversely, the microbiome in sandy clay soils can inhibit the growth of *B. striata*, while increasing the accumulation of militarine in tubers. Our findings provide preliminary insights into how soil properties and microbial communities can influence *B. striata* growth and synthesis of specific secondary metabolites, which may help guide efforts to optimize cultivation.

## Data Availability Statement

The datasets presented in this study can be found in online repositories. The names of the repository/repositories and accession number(s) can be found below: https://submit.ncbi.nlm.nih.gov/subs/bioproject/SUB11365206/overview.

## Author Contributions

CHX designed the experiments, analyzed the data, and contributed to the final version of the manuscript. TZ designed the experiments, supervised the study, and contributed to the final version of the manuscript. XZ performed experiments and contributed to the final version of the manuscript. CYX performed experiments, analyzed the data, and wrote the first draft of the manuscript. JZ, CY, WJ, JX, and YZ contributed to the final version of the manuscript. All authors reviewed and approved the manuscript before submission.

## Conflict of Interest

The authors declare that the research was conducted in the absence of any commercial or financial relationships that could be construed as a potential conflict of interest.

## Publisher’s Note

All claims expressed in this article are solely those of the authors and do not necessarily represent those of their affiliated organizations, or those of the publisher, the editors and the reviewers. Any product that may be evaluated in this article, or claim that may be made by its manufacturer, is not guaranteed or endorsed by the publisher.

## Abbreviations

A-Ca: available calcium
A-Cu: available copper
A-Fe: available iron
A-K: available potassium
A-Mg: available magnesium
A-Mn: available manganese
A-P: available phosphorus
A-Zn: available zinc
CK: control group
CMTS: clay-microbiota transplanted soil
DTPA: diethylene triamine penta-acetic acid
LMTS: loam-microbiota transplanted soil
SOC: soil organic carbon
SOM: soil organic matter
T-Ca: total calcium
T-Cu: total copper
T-Fe: total iron
T-K: total potassium
T-Mg: total magnesium
T-Mn: total manganese
T-N: total nitrogen
T-P: total phosphorus
T-Zn: total zinc.
